# Bilateral versus unilateral botulinum toxin injections for chronic anal fissure: a randomised trial

**DOI:** 10.1007/s10151-018-1821-2

**Published:** 2018-07-18

**Authors:** S. A. Pilkington, R. Bhome, R. E. Welch, F. Ku, C. Warden, S. Harris, J. Hicks, C. Richardson, T. C. Dudding, J. S. Knight, A. T. King, A. H. Mirnezami, N. E. Beck, P. H. Nichols, K. P. Nugent

**Affiliations:** 1 Department of Colorectal Surgery, Southampton General Hospital, University Hospitals Southampton NHS Trust, Southampton, UK; 2 Academic Surgical Unit, University of Southampton, Level C South Academic Block, Southampton General Hospital, Southampton, SO16 6YD UK; 3 School of Medicine, University of Southampton, Southampton, UK; 4 Department of Colorectal Surgery, University of Cape Town, Groote Schuur Hospital, Cape Town, South Africa; 5 Primary Care and Population Studies Unit, University of Southampton, Southampton General Hospital, Southampton, UK

**Keywords:** Anal fistula, Botulinum toxin, Injection

## Abstract

**Background:**

Botulinum toxin injected into the internal anal sphincter is used in the treatment of chronic anal fissure but there is no standardised technique for its administration. This randomised single centre trial compares bilateral (either side of fissure) to unilateral injection.

**Methods:**

Participants were randomised to receive bilateral (50 + 50 units) or unilateral (100 units) Dysport^®^ injections into the internal anal sphincter in an outpatient setting. Injection-related pain assessed by visual analogue scale was the primary outcome measure. Secondary outcomes were healing rate, fissure pain, incontinence, and global health scores.

**Results:**

Between October 2008 and April 2012, 100 patients with chronic anal fissure were randomised to receive bilateral or unilateral injections. Injection-related pain was comparable in both groups. There was no difference in healing rate. Initially, there was greater improvement in fissure pain in the bilateral group but at 1 year the unilateral group showed greater improvement. Cleveland Clinic Incontinence score was lower in the unilateral group in the early post-treatment period and global health assessment (EuroQol EQ-VAS) was higher in the unilateral group at 1 year.

**Conclusions:**

Injection-related pain was similar in bilateral and unilateral injection groups. Unilateral injection was as effective as bilateral injections in healing and improving fissure pain without any deterioration in continence.

**Electronic supplementary material:**

The online version of this article (10.1007/s10151-018-1821-2) contains supplementary material, which is available to authorized users.

## Introduction

Anal fissures are a common cause of anal pain. Most fissures will heal spontaneously but those that persist beyond 6 weeks are classified as chronic anal fissures (CAF). The aetiology of this disease is unclear, but hypertonia of the internal anal sphincter (IAS) is frequently present and may be sufficient to cause ischemia, resulting in persistent ulceration [1]. Treatment is aimed at reducing spasm of the IAS, relieving pain and promoting healing of the ulcer. The most effective method for CAF healing is lateral sphincterotomy [2]. Although this procedure is associated with healing rates of greater than 90% [3, 4], there is permanent weakening of the IAS and this may lead to incontinence of gas (6–9%), liquid (6–8%) and solid stool (1%) [5, 6]. This has resulted in enthusiasm for “chemical sphincterotomy” using pharmacological agents, including topical application of glyceryl trinitrate (GTN) or calcium channel blockers, and injection of botulinum toxin (BT) [7–9]. Although non-permanent, this causes a lowering of resting pressure for a temporary period allowing healing of the fissure. Success rates (65–75%) are significantly lower than those associated with surgical sphincterotomy [2, 10].

BT is an endopeptidase which blocks acetylcholine release at the neuromuscular junction. However, in the treatment of anal fissure the main effect of BT is by blockade of sympathetic (noradrenaline mediated) neural activity [11]. Dysport^®^ and Botox^®^ formulations of BT have the same efficacy and tolerability [12]. The efficacy of BT has been shown to be associated with total dose but not specifically to injection site or number of injections [13]. The overall healing rate with BT injection has been estimated at 65% [14].

Logically, a single injection of BT into this sensitive area is likely to be more tolerable than two injections; however, most studies in the literature use bilateral injections. One study used a single needleless injection of Botox^®^ with a novel delivery system at the site of the fissure [15]. Healing rates and reduction of resting pressure similar to that observed with conventional injection techniques were observed.

The aim of this study was to compare bilateral and unilateral BT injections for CAF. The primary outcome was pain associated with injection. Secondary outcomes were healing rate, fissure pain, anal incontinence and quality of life.

## Materials and methods

All patients who attended the CAF clinic at University Hospitals Southampton NHS Trust between October 2008 and April 2012 were invited to participate. Inclusion criteria were symptomatic anal fissures which had persisted for 6 weeks or longer and failed to respond to topical nitrate or calcium antagonist. Participants had to be over the age of 18 years with capacity to give informed written consent. Exclusion criteria were pregnancy, a history of inflammatory bowel disease, or an allergy to BT. The study protocol was approved by the regional ethics committee and assigned trial registration number NRES 08/H0501/50.

Participants were allocated randomly to treatment arms by a computer generated randomisation sequence. BT treatment was administered in the outpatient clinic with no local anaesthesia or sedation. Patients were placed in the left lateral position. Palpation of the intersphincteric groove was used to guide the injection. Injection sites were cleaned with ChoraPrep^®^. A 30-gauge needle was used for delivery of BT into the IAS. Patients in the bilateral injection group received 50 units of Dysport^®^ at both 3 and 9 O’clock positions. In the unilateral injection group, participants received a single injection of 100 units of Dysport^®^ into the IAS at 3 O’clock. It should be noted that 100 units Dysport^®^ is equivalent to 50 units Botox^®^.

Injection-related pain (VAS_injection_) and fissure pain (VAS_fissure_) were assessed on a 100 mm horizontal visual analogue scale with “no pain” (0 mm) at one end and “worst imaginable pain” (100 mm) at the other end. Patients also completed a baseline questionnaire, which included the Cleveland Clinic Incontinence (CCI) score [16], EQ-5D health profile [17] and EQ-VAS global assessment of health [18]. Anorectal manometry and clinical examination were conducted prior to injection. Follow-up was at 2, 8, 24 and 52 weeks using postal questionnaires to assess VAS_fissure_, CCI score, EQ-5D and EQ-VAS. Where questionnaires were not returned, patients were contacted to maximise returns. Patients were clinically reviewed until asymptomatic or referred for further treatment. Healing was defined as resolution of symptoms such that no further intervention was required.

### Statistical analysis

A sample size calculation was made based on an audit of VAS_injection_ associated with standard bilateral BT injections in eight patients (mean score 40.6 ± 21.7 mm). It was expected that one injection would be half as painful as two injections. In the audit this would be a reduction in pain score of 15 mm, or from an average of 40–25 mm. Using nQuery Advisor and the group means of 40 and 25 mm, a common standard deviation of 22 mm, standard levels of 5% significance with a 2-sided test and 80% power, a sample size of 35 patients per group was needed. Assuming a recruitment rate of 70%, it was estimated that 100 patients would need to be invited to participate and that this would take approximately 2 years.

SPSS version 21.0 and Confidence Interval Analysis version 2.2.1 were used for statistical analysis. For continuous variables the distribution was assessed using histograms. Normally distributed variables were analysed using *t* tests (paired and unpaired). Non-parametric variables were analysed using Mann–Whitney *U* test. Discrete variables were analysed using 2 × 2 contingency tables (Chi-squared test). Data is presented as mean (SD) unless otherwise stated.

## Results

**Fig. 1:**
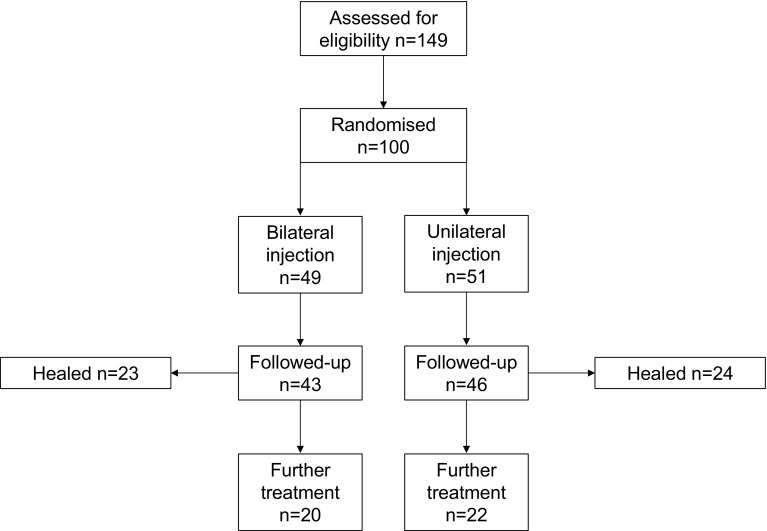
Consolidated Standards of Reporting Trials (CONSORT) flow diagram of randomised trial comparing bilateral and unilateral botulinum toxin injection for chronic anal fissure

In the study period, 149 patients were invited to participate in the study. A total of 100 patients were recruited and randomised (Fig. 1). Forty-nine had bilateral injections and 51 unilateral. Both groups had comparable demographic and clinical characteristics (Table 1). Cumulative questionnaire return rates were 60 and 51% for bilateral and unilateral injection groups respectively (*p* = 0.119; Table 2). It should be noted that the 2-week questionnaire was implemented after the first 26 patients had been recruited to the study, which accounts for the discrepancy in questionnaire uptake at this time point.

**Table 1:** Demographic and clinical characteristics at baseline

	Bilateral (*n* = 49)	Unilateral (*n* = 51)
Age (years)		
Mean (SD)	41.6 (13.2)	39.8 (16.0)
Range	21–74	19–80
Sex		
*M*	22 (44.9%)	19 (37.3%)
*F*	27 (55.1%)	32 (62.7%)
CAF site		
Posterior	32 (65.3%)	29 (56.9%)
Anterior	12 (24.5%)	18 (35.3%)
Both	3 (6.1%)	2 (3.9%)
Other	2 (4.1%)	1 (2.0%)
Not recorded	0	1 (2.0%)
Sentinel tag		
Yes	14 (28.6%)	10 (19.6%)
No	35 (71.4%)	41 (80.4%)
Pain		
Yes	49 (100%)	50 (98.0%)
No	0	1 (2.0%)
Bleeding		
Yes	37 (75.5%)	41 (80.4%)
No	12 (24.5%)	10 (19.6)
Symptom duration (months)	18 (10–66)	24 (12–59)
VAS_fissure_ (mm)	49.8 (26.3)	54.4 (24.9)
CCI	3.3 (2.6)	3.2 (3.5)
EQ-VAS	77.5 (16.2)	77.1 (16.3)

*CAF* chronic anal fissure, *VAS* visual analogue scale, *CCI* Cleveland Clinic Incontinence score, *EQ* EuroQol

**Table 2:** Questionnaire uptake at each follow-up interval in patients having bilateral and unilateral BT injections

Follow-up interval (weeks)	Bilateral	Unilateral
2	23/36 (64%)	19/38 (50%)
8	27/49 (55%)	31/51 (61%)
24	26/49 (53%)	22/51 (43%)
52	33/49 (67%)	26/51 (51%)
Total	109/183 (60%)	98/191 (51%)

*BT* botulinum toxin

Chi-squared test *p* = 0.119 for cumulative return rate. Two-week questionnaire introduced after first 26 patients had been recruited

The median VAS_injection_ was 25.5 (10.8–47.4) and 27.7 (12.3–50.8) mm in bilateral and unilateral groups respectively (*p* = 0.705; Mann–Whitney *U* test), demonstrating no significant difference in injection-related pain.

There was no difference in healing between bilateral and unilateral injection with rates of 53.5 and 52.2% respectively (Chi-squared test; *p* = 0.901). There was no difference in healing between posterior and anterior fissures (Chi-squared test; *p* = 0.637; data not shown). Similarly, there was no difference between symptom duration and healing (Chi-squared test; *p* = 0.742; data not shown).

VAS_fissure_ for both groups was significantly lower at all follow-up intervals compared to baseline, except at 2 weeks in the unilateral group (Supplementary Table 1). The absolute reduction in VAS_fissure_ from baseline was significantly worse in the unilateral compared to bilateral group at 2 weeks, not significantly different at 2 and 6 months, and significantly better at 1 year (Table 3).

**Table 3:** Change in fissure pain (VAS_fissure_) at follow-up compared to baseline in patients having bilateral and unilateral BT injections

Follow-up (weeks)	Δ VAS_fissure_ bilateral (mm)	Δ VAS_fissure_ unilateral (mm)	Absolute difference	p*
2	− 23.0 (24.3)	− 7.4 (22.3)	− 15.6 [− 30.3 to − 0.9]	**0.038**
8	− 17.5 (30.2)	− 12.9 (32.0)	− 4.5 [− 21.0 to 11.9]	0.584
24	− 15.7 (23.3)	− 22.6 (28.1)	6.9 [− 8.4 to 22.2]	0.369
52	− 22.3 (30.0)	− 39.1 (32.2)	16.8 [0.5 to 33.2]	**0.044**

Significant *p* values are given in bold

*VAS* visual analogue scale

*Independent *t* test. Absolute difference calculated as Δ_bilateral_ − Δ_unilateral_

In the bilateral group, CCI scores were significantly increased compared to baseline at 2 weeks (+ 1.9 [CI 0.7–3.1]) and 2 months (+ 1.6 [CI 0.4–2.7]) but not different at 6 months and 1 year. In the unilateral group there was no increase in CCI score at any of the follow-up intervals (Supplementary Table 2). A direct comparison showed a significantly lower CCI score at 2 weeks in the unilateral compared to bilateral group, which reached parity from 2 months onwards (Table 4).

**Table 4:** CCI score at different follow-up intervals in patients having bilateral and unilateral BT injections

Follow-up (weeks)	CCI bilateral	CCI unilateral	p*
2	5.0 (3.0)	3.1 (2.6)	**0.036**
8	4.3 (3.0)	4.2 (4.5)	0.922
24	4.2 (1.5)	3.9 (4.7)	0.768
52	3.5 (2.6)	3.7 (2.9)	0.811

Significant *p* value is given in bold

*CCI* Cleveland Clinic Incontinence score, *BT* botulinum toxin

*Independent *t* test

No significant differences were found in EQ-5D scores at any follow-up interval compared to baseline for either group (Supplementary Table 3). However, at 1 year, the unilateral group had a significantly higher EQ-VAS score compared to baseline (+ 7.3 [CI 0.8–13.7]), indicating an improvement in overall health state (Supplementary Table 4). A direct comparison showed a higher EQ-VAS in the unilateral compared to bilateral group at 1 year (Table 5).

**Table 5:** Global health assessment (EQ-VAS) at different follow-up intervals in patients having bilateral and unilateral BT injections

Follow-up (weeks)	EQ-VAS bilateral	EQ-VAS unilateral	p*
2	72.8 (21.1)	76.5 (18.2)	0.555
8	76.8 (21.2)	75.6 (20.4)	0.827
24	75.0 (24.2)	82.6 (14.6)	0.209
52	78.4 (19.0)	87.2 (10.3)	**0.037**

Significant *p* value is given in bold

*EQ* EuroQol, *VAS* visual analogue scale, *BT* botulinum toxin

*Independent *t* test. An increase in EQ-VAS represents an improvement in health state

**Fig. 2:**
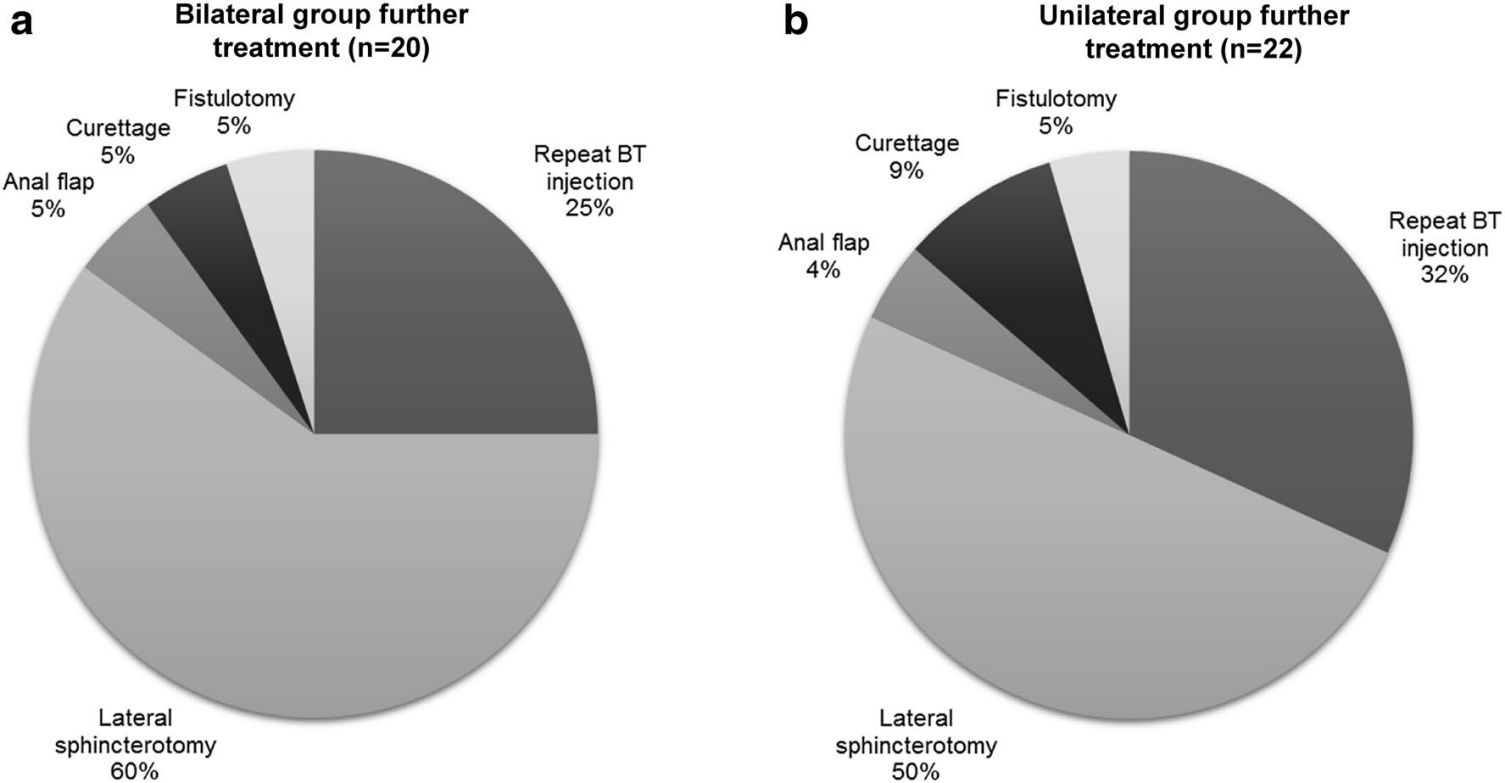
Further treatment in patients failing to heal in bilateral (**a**) and unilateral (**b**) injection groups

Of the patients who failed to heal, surgical intervention was required in 15/20 cases (75%) in the bilateral group and 15/22 cases (68%) in the unilateral group (Fig. 2). The rate of surgical intervention in general and lateral internal sphincterotomy in particular, was not significantly different between groups (*p* = 0.739 and *p* = 0.551 respectively).

Twelve patients had repeat BT injection administered in the same manner as their initial randomisation: 5 in the bilateral injection group and 7 in the unilateral group. Healing rate after repeat BT was 2/5 (40%) in the bilateral group and 3/7 (43%) in the unilateral group. Outcomes for these patients are listed in Supplementary Table 5.

There were three complications in total. One patient in the bilateral group developed an infected haematoma and required oral antibiotics. Two patients (one from each group) developed fistula-in-ano requiring fistulotomy.

## Discussion

This randomised trial showed that there was no difference in injection-related pain for bilateral and unilateral BT (Dysport^®^) injections. Unilateral injections were as effective as bilateral injections in inducing healing. However, unilateral injection was more effective than bilateral injection in improving fissure pain at 1 year. This was associated with an improvement in measurable health state (EQ-VAS) at this time point. Furthermore, unilateral injection had no effect on anal incontinence, whereas bilateral injection led to increased CCI score in the early post-treatment period. Both bilateral and unilateral injections were safe in the outpatient setting.

The dose and site of BT injection has not been standardised. Injection into or on either side, close to the fissure has been described, but local fibrosis and scarring may reduce the effectiveness [19]. In fact, up to eight injection sites have been described, but most studies use bilateral injections [20]. A single unilateral injection into this sensitive area was thought to be more tolerable for patients than two injections. However, in the present study there was no significant difference in injection-related pain in the two groups. From our observations, patients did not find the injections as painful as they had anticipated but this was not formally assessed. Moreover, this was a non-blinded study; therefore, participant’s knowledge of the number of injections they were to receive may have influenced pain scores. The experience of pain is subjective but both treatment groups rated the pain associated with injection as markedly lower than pain from their fissure.

To the best of our knowledge there are no previous studies comparing injection-related pain for bilateral versus unilateral injection of BT. However, Festen et al. [21] demonstrated a mean VAS_injection_ of 58 mm with bilateral injections using Botox^®^. In this study, the needle was guided into the IAS with the index finger in the anal canal. This is likely to have increased the pain experienced by the patient due to direct pressure on the fissure. This may explain why the pain scores were more than twice as high as those recorded in the present study. We refrained from performing digital rectal examination at the time of injection as this had already been carried out previously. The low pain scores associated with BT injection in this study emphasise the feasibility of performing BT treatment in the outpatient setting.

It is unclear why bilateral injections were more effective at improving fissure pain initially (2 weeks) and unilateral injections were more effective at 1 year (Table 3; Supplementary Table 1). The difference in efficacy at 1 year is unlikely to be related to degree of denervation because the duration of action of BT is typically 2–3 months [22, 23]. Moreover, it could be argued that fissure pain is skewed in the follow-up period by patients who have healed completely (who should register a VAS_fissure_ of zero). However, the healing rate in both groups was comparable and this bias was expected to affect both groups equally.

With regard to incontinence, bilateral injections may have targeted a greater area of internal sphincter, causing not only an increased reduction in anal tone (associated with greater improvement in pain scores), but a resultant reduction in resting pressure which then led to increased CCI scores (above baseline) in the early post-treatment period. Absolute increase in CCI score above baseline in the bilateral group was between 1.5 and 1.9 points, up to 2 months following treatment (Supplementary Table 2). This is not insignificant and equates to a patient who was rarely incontinent becoming usually incontinence to either solid stool, liquid or gas.

EQ-VAS score improved (increased) significantly above baseline only in the unilateral group at the 1-year follow-up interval (Supplementary Table 4). EQ-5D also improved (decreased) in this group at 1 year, but did not reach significance (Supplementary Table 3). Interestingly, this correlated with the greatest improvement in VAS_fissure_ (− 39.1 mm), also recorded at 1 year in the unilateral group, suggesting that fissure pain may contribute significantly to the global health of these patients, and that VAS_fissure_ needs to be reduced in the order of 70% before any improvement in perceived health is registered (Table 3).

The present study is subject to a number of limitations. The overall postal questionnaire return rates were 60 and 51% for bilateral and unilateral injection groups, respectively, and may, therefore, be a potential confounding factor. A well-known limitation of visual analogue scales is end-of-scale bias where participants are less likely to choose values at extreme ends. Nonetheless, this method has been extensively validated and has been used previously for this particular disease [24, 25]. This was a non-blinded trial and patients were aware which group they were randomised to, which may have introduced reporting bias. Finally, the study was powered for the primary outcome of injection-related pain and may not necessarily be suitably powered for other outcomes. Results should be interpreted with these limitations in mind.

## Conclusions

There was no difference in injection-related pain for bilateral and unilateral BT injections. However, unilateral BT injection was equally effective in inducing healing, more effective in improving fissure pain at 1 year and less detrimental to continence in the short term. This method of BT administration should considered when formulating guidelines on the management of CAF.

## Electronic supplementary material

Below is the link to the electronic supplementary material.

Supplementary material 1 (DOCX 26 KB)
