# Supplementary material for: Bilateral versus unilateral botulinum toxin injections for chronic anal fissure: a randomised trial
Source: Tech Coloproctol. 2018 Jul 18;22(7):545–51. doi: 10.1007/s10151-018-1821-2 (PMC6097731; doi:10.1007/s10151-018-1821-2)
Supplement: Supplementary file 1 — Supplementary material 1 (DOCX 26 KB) [file 10151_2018_1821_MOESM1_ESM.docx]

**Supplementary Material**

**Bilateral versus unilateral botulinum toxin injections for chronic anal fissure: A randomised trial**

SA Pilkington, R Bhome, RE Welch, F Ku, C Warden, S Harris, J Hicks, C Richardson, TC Dudding, JS Knight, AT King, AH Mirnezami, NE Beck, PH Nichols, KP Nugent

**Supplementary figures: 0**

**Supplementary tables: 5**

**Supplementary Table 1. A comparison of fissure pain (VAS_fissure_) at different follow-up intervals compared to baseline in patients having bilateral and unilateral BT injections.**

|  | **Follow-up (weeks)** | **Number of patients** | **Baseline VAS_fissure_ (mm)** | **Follow-up VAS_fissure_ (mm)** | **Absolute difference** | ***P^*^*** |
| --- | --- | --- | --- | --- | --- | --- |
| **Bilateral injections** | 2 | 23 | 53.5 (28.6) | 30.5 (23.0) | -23.0 [-33.5 to -12.5] | **<0.001** |
|  | 8 | 27 | 47.2 (29.6) | 29.8 (26.9) | -17.5 [-29.4 to -5.5] | **0.006** |
|  | 24 | 24 | 46.9 (28.6) | 31.2 (28.2) | -15.7 [-25.6 to -5.9] | **0.003** |
|  | 52 | 32 | 49.8 (26.3) | 27.6 (27.8) | -22.3 [-33.0 to -11.5] | **<0.001** |
| **Unilateral injection** | 2 | 19 | 48.7 (28.0) | 41.3 (23.1) | -7.4 [-18.1 to 3.3] | 0.165 |
|  | 8 | 31 | 54.4 (24.9) | 41.4 (27.0) | -13.0 [-24.7 to -1.2] | **0.032** |
|  | 24 | 22 | 52.9 (24.6) | 30.3 (25.4) | -22.6 [-35.1 to -10.1] | **0.001** |
|  | 52 | 26 | 56.6 (28.8) | 17.5 (23.5) | -39.1 [-52.1 to -26.1] | **<0.001** |

*Paired t-test. *VAS – visual analogue scale; BT – botulinum toxin*

**Supplementary Table 2. A comparison of CCI score at different follow-up intervals compared to baseline in patients having bilateral and unilateral BT injections.**

|  | **Follow-up (weeks)** | **Number of patients** | **Baseline CCI** | **Follow-up CCI** | **Absolute difference** | ***P^*^*** |
| --- | --- | --- | --- | --- | --- | --- |
| **Bilateral injections** | 2 | 23 | 3.2 (2.5) | 5.0 (3.0) | 1.9 [0.7 to 3.1] | **0.004** |
|  | 8 | 27 | 2.7 (2.6) | 4.3 (3.0) | 1.6 [0.4 to 2.7] | **0.009** |
|  | 24 | 24 | 2.8 (2.7) | 4.2 (1.5) | 1.5 [-0.1 to 3.0] | 0.058 |
|  | 52 | 32 | 3.3 (2.6) | 3.5 (2.6) | 0.1 [-0.9 to 1.2] | 0.811 |
| **Unilateral injection** | 2 | 19 | 2.8 (4.0) | 3.1 (2.6) | 0.3 [-1.8 to 2.4] | 0.754 |
|  | 8 | 31 | 3.2 (3.5) | 4.2 (4.5) | 1.1 [-0.3 to 2.4] | 0.121 |
|  | 24 | 22 | 2.6 (2.6) | 3.9 (4.7) | 1.3 [-0.54 to 3.1] | 0.160 |
|  | 52 | 26 | 3.9 (4.3) | 3.7 (2.9) | -0.2 [-1.8 to 1.4] | 0.803 |

*Paired t-test. *CCI – Cleveland Clinic Incontinence score; BT – botulinum toxin*

**Supplementary Table 3. A comparison of health profile (EQ-5D) at different follow-up intervals in patients having bilateral and unilateral BT injections.**

|  | **Follow-up (weeks)** | **Number of patients** | **Baseline EQ-5D** | **Follow-up EQ-5D** | **Absolute difference** | ***P^*^*** |
| --- | --- | --- | --- | --- | --- | --- |
| **Bilateral injections** | 2 | 23 | 6.8 (1.6) | 6.6 (1.8) | -0.2 [-0.8 – 0.42] | 0.549 |
|  | 8 | 26 | 6.5 (1.3) | 6.7 (1.7) | -0.2 [-0.4 – 0.7] | 0.548 |
|  | 24 | 24 | 6.6 (1.3) | 6.2 (1.5) | -0.4 [-1.0 – 0.4] | 0.233 |
|  | 52 | 33 | 6.6 (1.3) | 6.3 (1.3) | -0.3 [-0.8 – 0.12] | 0.148 |
| **Unilateral injection** | 2 | 19 | 6.4 (1.3) | 6.5 (1.2) | 0.1 [-0.3 – 0.5] | 0.578 |
|  | 8 | 30 | 6.5 (1.1) | 6.6 (1.4) | 0.1 [-0.4 – 0.4] | 0.861 |
|  | 24 | 22 | 6.3 (0.9) | 6.1 (0.9) | -0.2 [-0.7 – 0.3] | 0.446 |
|  | 52 | 26 | 6.5 (1.3) | 6.1 (1.1) | -0.5 [-1.0 – 0.1] | 0.083 |

*Paired t-test. A decrease in EQ-5D represents an improvement in health state. *BT – botulinum toxin*

**Supplementary Table 4. A comparison of global health assessment (EQ-VAS) at different follow-up intervals compared to baseline in patients having bilateral and unilateral BT injections.**

|  | **Follow-up (weeks)** | **Number of patients** | **Baseline EQ-VAS** | **Follow-up EQ-VAS** | **Absolute difference** | ***P^*^*** |
| --- | --- | --- | --- | --- | --- | --- |
| **Bilateral injections** | 2 | 22 | 71.3 (22.6) | 72.8 (21.1) | 1.5 [-5.3 to 8.2] | 0.657 |
|  | 8 | 27 | 71.5 (26.6) | 76.8 (21.2) | 5.3 [-1.9 to 12.4] | 0.140 |
|  | 24 | 24 | 71.9 (23.5) | 75.0 (24.2) | 3.1 [-5.7 to 11.9] | 0.417 |
|  | 52 | 33 | 77.5 (16.2) | 78.4 (19.0) | 0.9 [-6.0 to 7.8] | 0.791 |
| **Unilateral injection** | 2 | 19 | 80.2 (17.1) | 76.5 (18.2) | -3.7 [-8.8 to 1.4] | 0.143 |
|  | 8 | 31 | 77.1 (16.3) | 75.6 (20.4) | -1.5 [-8.2 to 5.2] | 0.654 |
|  | 24 | 22 | 79.1 (15.3) | 82.6 (14.6) | 3.5 [-6.0 to 13.0] | 0.450 |
|  | 52 | 26 | 79.9 (15.1) | 87.2 (10.3) | 7.27 [0.8 to 13.7] | **0.029** |

*Paired t-test. An increase in EQ-VAS represents an improvement in health state. *BT – botulinum toxin*

**Supplementary Table 5. Outcome of patients who underwent repeat BT injection.**

| **Repeat bilateral injection (n=5)** | **Repeat unilateral injection (n=7)** |
| --- | --- |
| - Healed (2) - Further BT injection (1) - Lateral sphincterotomy (1) - Lost to follow-up (1) | - Healed (3) - Lateral sphincterotomy (3) - Lost to follow-up (1) |

*BT – botulinum toxin*
